# Antibody-based screening of cell wall matrix glycans in ferns reveals taxon, tissue and cell-type specific distribution patterns

**DOI:** 10.1186/s12870-014-0362-8

**Published:** 2015-02-18

**Authors:** Olivier Leroux, Iben Sørensen, Susan E Marcus, Ronnie LL Viane, William GT Willats, J Paul Knox

**Affiliations:** Pteridology, Department of Biology, Ghent University, K.L. Ledeganckstraat 35, Ghent, B-9000 Belgium; Department of Plant Biology and Biotechnology, Copenhagen University, Thorvaldsensvej 40, Frederiksberg, 1871 Denmark; Department of Plant Biology, Cornell University, Ithaca, NY 14853 USA; Centre for Plant Sciences, Faculty of Biological Sciences, University of Leeds, Leeds, LS2 9JT UK

**Keywords:** Cell wall evolution, Homogalacturonan, Arabinan, Galactan, Xyloglucan, Xylan, Mannan, Mixed-linkage glucan, Sclerenchyma

## Abstract

**Background:**

While it is kno3wn that complex tissues with specialized functions emerged during land plant evolution, it is not clear how cell wall polymers and their structural variants are associated with specific tissues or cell types. Moreover, due to the economic importance of many flowering plants, ferns have been largely neglected in cell wall comparative studies.

**Results:**

To explore fern cell wall diversity sets of monoclonal antibodies directed to matrix glycans of angiosperm cell walls have been used in glycan microarray and *in situ* analyses with 76 fern species and four species of lycophytes. All major matrix glycans were present as indicated by epitope detection with some variations in abundance. Pectic HG epitopes were of low abundance in lycophytes and the CCRC-M1 fucosylated xyloglucan epitope was largely absent from the Aspleniaceae. The LM15 XXXG epitope was detected widely across the ferns and specifically associated with phloem cell walls and similarly the LM11 xylan epitope was associated with xylem cell walls. The LM5 galactan and LM6 arabinan epitopes, linked to pectic supramolecules in angiosperms, were associated with vascular structures with only limited detection in ground tissues. Mannan epitopes were found to be associated with the development of mechanical tissues. We provided the first evidence for the presence of MLG in leptosporangiate ferns.

**Conclusions:**

The data sets indicate that cell wall diversity in land plants is multifaceted and that matrix glycan epitopes display complex spatio-temporal and phylogenetic distribution patterns that are likely to relate to the evolution of land plant body plans.

**Electronic supplementary material:**

The online version of this article (doi:10.1186/s12870-014-0362-8) contains supplementary material, which is available to authorized users.

## Background

The colonisation of land was a major event in the history of plants. Subsequent widespread ecological radiation and diversification was directed by complex interactions involving the interplay between morpho-anatomical and physiological adaptations of plants and the physical and chemical changes in their environment. Many adaptations facilitated terrestrial colonisation and survival, including anchorage and water uptake, mechanical support, water transport, protection against desiccation and UV-irradiance, as well as reproduction in absence of water [1]. Specialised tissues and cell types, especially in the vegetative body, emerged and contributed to the structural complexity of plants. As the architecture and properties of cell walls largely determine tissue/organ structure and function and consequently overall morphology, they must have played a fundamental role in the evolution and differentiation of complex body plans.

By the end of the 19^th^ century, the combined efforts of many plant anatomists led to an increased knowledge of the anatomical complexity of land plants, resulting in the distinction of tissues and cell types that are still recognised today [2]. These tissues are composed of cells with walls that are classed as either primary cell walls that prevent cell bursting and regulate cell expansion, or non-extendable secondary cell walls, restricted to certain cell types, which have mechanical properties resisting external forces that would lead to cell collapse. Both types of walls are structurally complex composites. In most primary cell walls a load bearing network of cellulose microfibrils is cross-linked and interspersed with complex sets of matrix glycans including those classed as hemicelluloses (xyloglucans, heteroxylans, heteromannans and mixed-linkage glucans) and the multi-domain pectic supramolecular polysaccharides [3,4]. Secondary cell walls are often reinforced with lignin and contain low amounts of pectins. Many cell wall components may display considerable heterogeneity, either in their molecular structure or in their spatio-temporal distribution in plant organs, tissues, cell-types and individual walls [3,5]. As wall components may be present in variable amounts in different cell walls at specific developmental stages, there is not always a clear distinction in molecular composition between primary and secondary cell walls [6]. Moreover, walls may be modified in response to environmental stress or pathogen attack [7] and even after cell death (e.g. postmortem lignification [8]).

Cell walls also display remarkable diversity at the taxonomical level as the presence and/or abundance of specific wall components may vary between the major plant lineages (e.g. [9-17]; see [18] for a brief overview). Analysis of the early diverging fern (s.l., monilophyta) *Equisetum* [19,20] has indicated structurally distinct cell walls that do not fit within either the type I or type II classification that had been developed for angiosperm cell walls [21,22]. Recently, a third mannan-rich (primary) cell wall type (cell wall type III), typical of ferns was reported [23]. Although broadly useful in reflecting major taxonomic distinctions in global compositional differences, classifications of cell wall types neglects variation in wall components between cell types within organs and most notably may not relate to all land plant species. In addition, little is known of how the range of polysaccharides found in primary and secondary cell walls relates to the evolution of specific cell wall functions and cell types.

**Figure 1:**
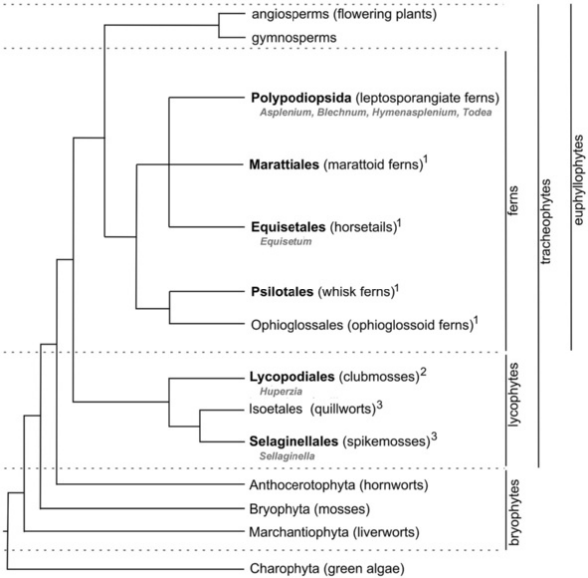
**Schematic tree showing the relationships among the major groups of land plants.** 1: eusporangiate ferns *s.l*.; 2: homosporous lycophytes; 3: heterosporous lycophytes. Representatives of the plant groups indicated in bold were sampled for this study (see Supplementary Figure 1). Genera represented in the immunofluorescence figures are indicated (grey). Adapted from [74,75].

To develop a deeper understanding of cell wall diversity within the context of tissues, cell types and individual walls in a group of land plants that has not been previously extensively studied, we carried out a glycan microarray analysis complemented with selected *in situ* immunolabelling of 76 fern species and 4 lycophytes species (Figure 1). Through extensive sampling within leptosporangiate ferns, and Aspleniaceae in particular, we aimed to identify tissue or cell type-specific distribution patterns of matrix glycan epitopes, but also explore variation in matrix glycan cell wall composition at family and species levels.

## Results and discussion

**Figure 2:**
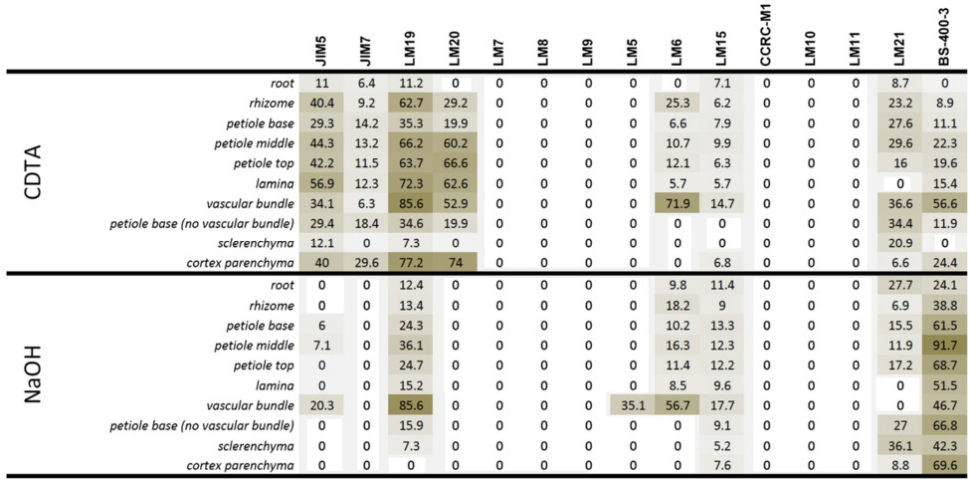
**Glycan microarray heatmap of CDTA and NaOH extracts of total organ or isolated tissue(s) of the leptosporangiate fern** ***Asplenium elliottii*** **.** The probes are listed at the top of the heatmap. References for probe specificity are listed in Table 1. Abbreviations: mAb: monoclonal antibody; HG: pectic homogalacturonan; AGP: arabinogalactan protein; XG: xyloglucan; Me: methyl-esterified.

**Figure 3:**
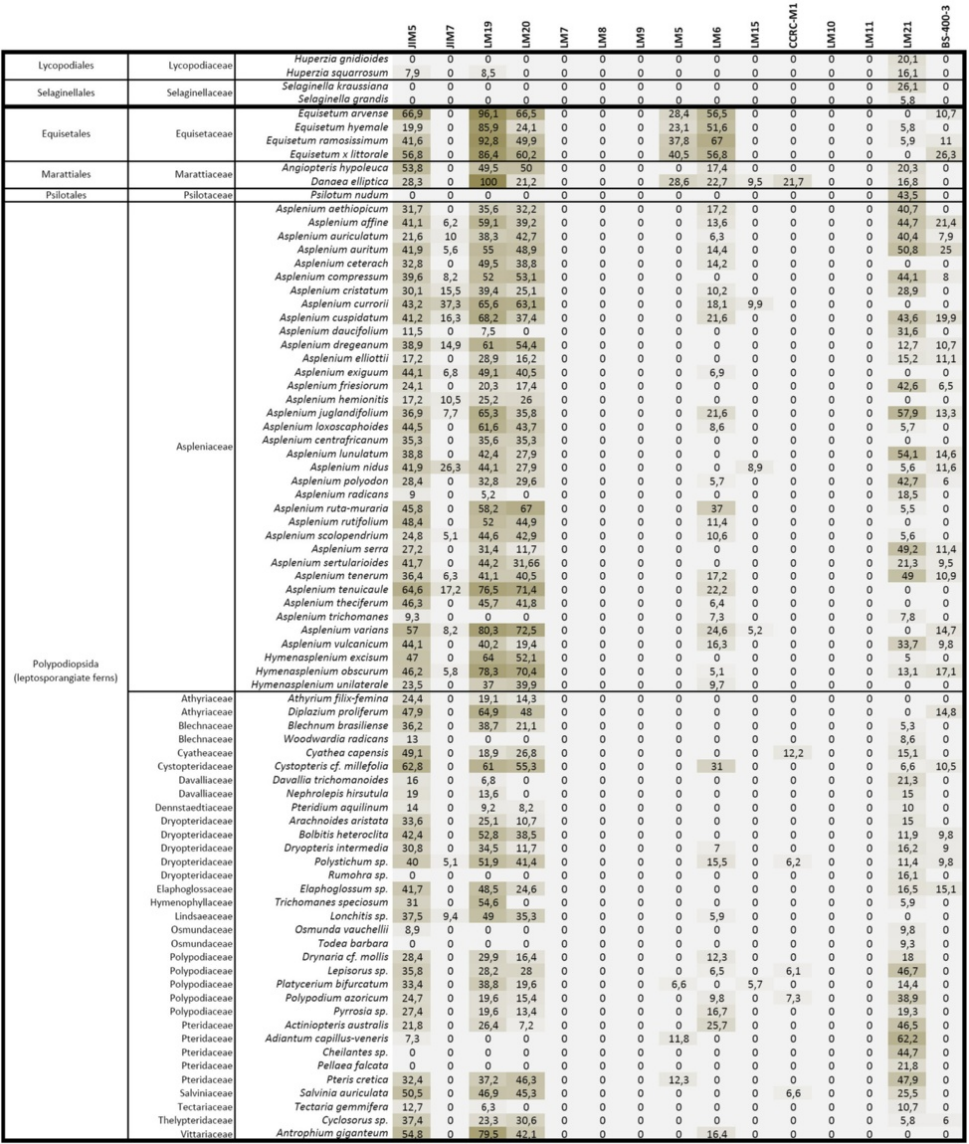
**Glycan microarray heatmap of CDTA extracts of fern or lycophyte petioles/stems.** References for probe specificity are listed in Table 1.

**Figure 4:**
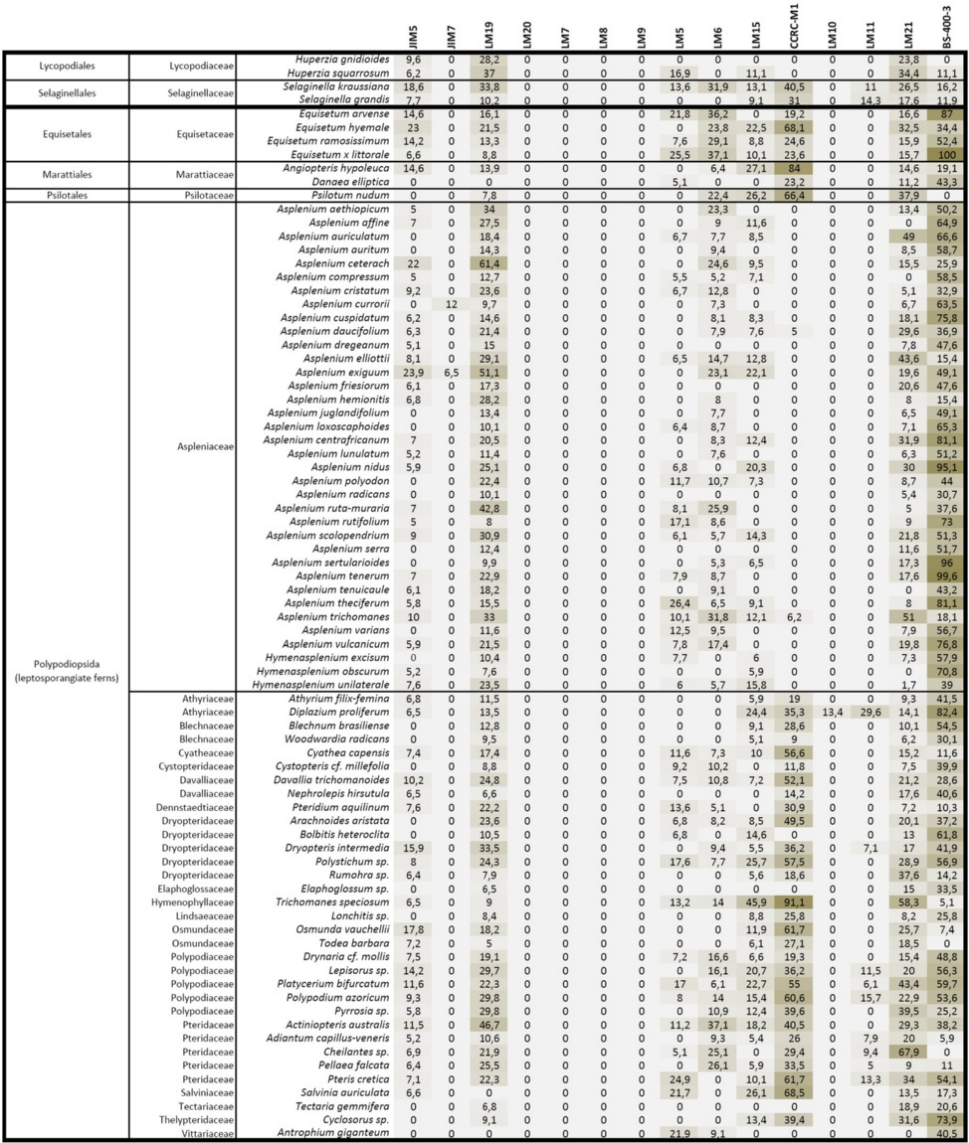
**Glycan microarray heatmap of NaOH extracts of fern or lycophyte petioles/stems.** References for probe specificity are listed in Table 1.

Interpretation of the glycan microarray analysis was approached from the perspective of cell wall polysaccharide classes and the results are presented as heatmaps (Figures 2, 3 and 4). An exploratory glycan microarray analysis of organs and tissues of the leptosporangiate fern *Asplenium elliotti* revealed considerable variation in the relative abundance of glycan epitopes among samples with most epitopes being detected in the petiole tissues (Figure 2). As our aim was to explore tissue-specific distribution of glycan epitopes across ferns we performed a broad-scale glycan microarray analysis by sampling only petiole bases (or stems in the case of *Huperzia*, *Selaginella*, *Psilotum* and *Equisetum*). The resulting heatmaps are shown in relation to both fern division and molecular probe class (Figures 3 and 4).

Variation in the dataset may reflect differences in developmental stage and health between plants, but also differences in extractability of specific components (e.g. lignification might hinder extraction of wall components) and tissue- and cell-type specific differences in cell wall composition. In several cases no binding of specific monoclonal antibodies (mAbs) above background was detected neither in the glycan microarray analysis, nor in the immunofluorescence analyses, indicating that the epitopes were not extracted, absent, or of (relatively) low-abundance. Therefore, if epitopes were not positively identified (indicated with “0” in the heatmaps) one cannot conclude that they are absent. Moreover, as we did not sample all organs and structures (including roots, rhizomes and laminae but also meristems and differentiating tissues) for each of the species studied, we can by no means state that certain epitopes are absent in the plant.

To understand the variation in epitope abundance we performed *in situ* immunolabelling experiments using the same antibodies as used for probing the glycan microarrays. As mAbs are epitope-specific and not polymer-specific, and, some epitopes might be masked by other wall components [24], we cannot draw any firm conclusions on general fern cell wall composition. However, immunofluorescence (IF) is a powerful tool to explore spatial patterns in glycan-epitope distribution, which is the main aim of this study.

Broad themes that became apparent in the glycan epitope analysis included the observation that the majority of the epitopes characterized in angiosperms were generally present across the assessed fern species. While we found no evidence for the presence of some epitopes including the LM7 homogalacturonan epitope that occurs at corners of intercellular spaces in angiosperms, the LM8 xylogalacturonan epitope that is detected in detaching cells and the LM9 feruloylated galactan epitope of Amaranthaceae cell walls, all other epitopes of cell wall matrix components were detected in variable (relative) amounts, and these are discussed below. As we can only show a selection of images, we chose to represent variation by selecting those images that provide most clarity with respect to general or very specific labelling patterns. In most cases we show magnifications of vascular bundles (typically xylem surrounded by phloem, pericycle and endodermis) or mechanical tissues (either sclerenchymatous or collenchymatous).

### Differential occurrence of pectic homogalacturonan (HG) epitopes in ferns

Homogalacturonan (HG) is the major pectic polysaccharide in angiosperms and a range of mAbs (e.g. JIM5, JIM7, LM19 and LM20) are available that recognize subtly different methyl-esterification patterns of this polymer [25-27]. HG is an abundant component of the primary cell walls of most angiosperms, except in the grasses where the total pectic content is low [22]. Studies have provided evidence for the presence of HGs in gymnosperms, ferns, lycophytes and charophycean green algae [9,28-30].

**Figure 5:**
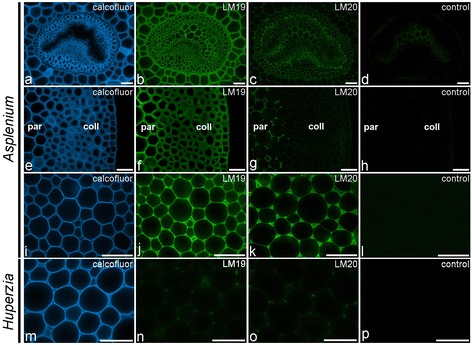
**Indirect immunofluorescence detection of homogalacturonan epitopes with low (LM19) and high (LM20) levels of esterification in fern petioles and lycophyte stems.** Calcofluor White fluorescence **(a, e, i, m)** shows the full extent of cell walls. **(a-d)** LM19 is detected in primary cell walls of the vascular bundle of *Asplenium rutifolium*, while LM20 is restricted to the middle lamellae and intercellular space corners. **(e–h)** The prevalence of the LM19 epitope over the LM20 epitope is apparent in parenchymatous and collenchymatous tissue of *Asplenium rutifolium*. **(i–l)** LM19 is detected in primary cell walls of *Asplenium daucifolium*, while LM20 is restricted to the middle lamellae and intercellular space corners. **(m–p)** LM19 and LM20 weakly bind to primary cell walls in the lycophyte *Huperzia squarrosum*. Abbreviations: par, parenchyma; coll, collenchymatous tissue. No primary antibody controls are provided **(d, h, l, p)**. Scale bars: 40 μm.

In the glycan microarray analysis pectic HG was widely detected (by JIM5, LM19 and LM20) in the CDTA-extracts of the majority of fern samples (Figures 2 and 3). The *in situ* distribution of two of the HG-directed mAbs was shown by IF (Figure 5). A distinctive feature of IF was that the LM19 epitope (low levels of methyl-esterification) was generally more abundant than the LM20 epitope (high levels of methyl-esterification). LM19 bound to primary cell walls, whereas LM20 had a more restricted binding pattern to the middle lamellae and intercellular space linings (Figure 5a–l); conversely to what is generally observed in angiosperm parenchyma [31]. The prevalence of the LM19 epitope over the LM20 epitope was also apparent in collenchymatous cell walls (Figure 5e–h). We obtained no evidence for the presence of the LM7 HG epitope (a specific methyl-esterification pattern) in any of the fern and lycophyte samples studied, although it has been reported in angiosperms [21] and green algae [28]. In the case of the lycophyte *Huperzia*, the eusporangiate whisk fern *Psilotum*, and some leptosporangiate ferns such as *Adiantum, Asplenium trichomanes* and *Davallia*, only low levels of pectic HG epitopes were detected in the CDTA-extracts. IF confirmed these results for *Huperzia* (Figure 5m–p), and further suggests that pectic HG might not be a major constituent of their cell walls or that these species have distinct cell wall architectures that hinder the extraction and/or detection of pectic homogalacturonans. In *Adiantum*, *Asplenium trichomanes* and *Pellea*, on the other hand, the cortical tissues are sclerified, and, as secondary cell walls generally contain no or only small amounts of pectins [31], a low HG content was to be expected.

### 1,5-arabinan and 1,4-galactan epitopes associated with specific tissues and/or cell types

Analysis of the pectic component rhamnogalacturan-I (RG-I) was performed by means of the arabinan and galactan-directed mAbs LM6 and LM5, respectively. Although 1,5-arabinans and 1,4-galactans are present in the complex heterogeneous pectic polymer RG-I [32], 1,5-arabinan may also be a constituent of arabinogalactan proteins [33]. RG-I is highly variable both in structure and occurrence within cell walls [34-37] and many have suggested that RG-I side chains exhibit developmentally-linked structural variation [33,38,39]. Both epitopes have been immunodetected in mature tissues of green algae [40], ferns [29,41] and angiosperms [37,42].

**Figure 6:**
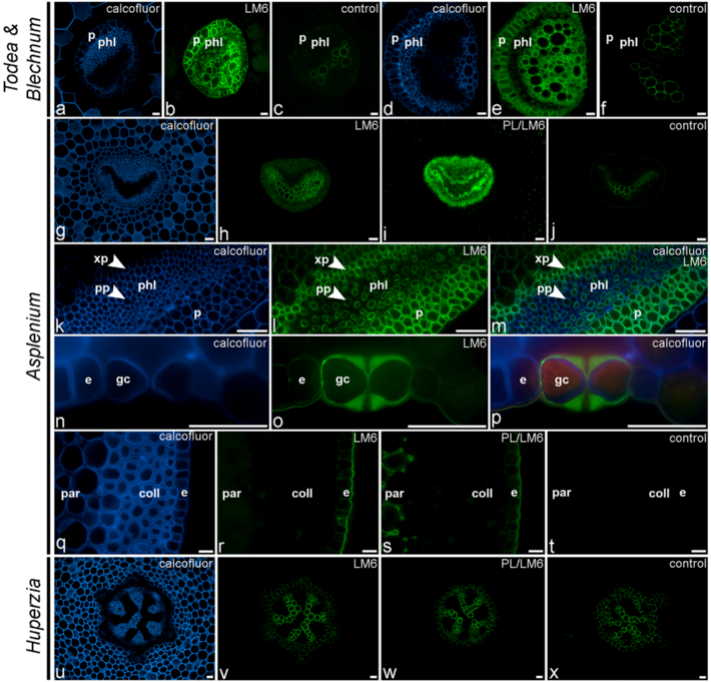
**Indirect immunofluorescence detection of the arabinan (LM6) epitope in transverse sections of fern petioles and lycophyte stems.** Calcofluor White fluorescence **(a, d, g, k, m, n, p, q, u)** shows the full extent of cell walls. **(a–c)** Detection of the LM6-epitope in parenchymatous cell types of vascular bundles of *Todea sp.* **(a–c)** and *Blechnum brasiliense* **(d–f)**. **(g–m)** Similar distribution pattern of the LM6-epitope is found in the vascular bundle **(g–j)** of *Asplenium theciferum*. Higher magnification **(k–m)** showing binding of LM6 to the cell walls of phloem parenchyma (pp), xylem parenchyma (xp) and pericycle (p). **(n–p)** LM6 binding to epidermal **(e)** cell walls, including the guard cell walls (gc) of stomata. **(q–t)** Detection of LM6 epitope in cortical parenchyma after pectate lyase (PL) treatment **(s)**. **(u–x)** LM6-epitope is not detected in the lycophyte *Huperzia squarrosum*, even after pectate lyase treatment **(w)**. Abbreviations: p, pericycle; phl, phloem; xp, xylem parenchyma; pp, phloem parenchyma; par, parenchyma; coll, collenchymatous tissue; e, epidermis. No primary antibody controls are provided **(c, f, j, t, x)**. Scale bars: 40 μm.

In the glycan microarray analysis the arabinan LM6-epitope was detected in the CDTA- and NaOH-extracts of most species, with relative high amounts in *Equisetum* (horsetails) and marattioid ferns, and absent in homosporous lycophytes (*Huperzia*) (Figure 2, 3 and 4). In the CDTA extracts of isolated vascular bundles of *A. elliottii* (Figure 2) the LM6 epitope was highly abundant relative to what was found for other tissues. Supportive IF showed consistent distribution patterns of the LM6-epitope among the leptosporangiate ferns (Figure 6), being specifically and strongly immunodetected in the phloem and xylem parenchyma, and the pericycle (Figure 6a–m), the parenchymatous cell types of the vascular bundle. LM6 also labelled epidermal cell walls, including the guard cell walls (Figure 6n–p). Arabinans have been reported to play key roles in determining guard cell wall flexibility in angiosperms [42], and their detection in guard cell walls of *Equisetum* [37] and other ferns such as *Asplenium* suggests that arabinans might have played an important role in the functional evolution of stomata. Pectate lyase pretreatment of sections prior to IF unmasked LM6-epitopes in the cortical parenchyma cell walls of many species including *Asplenium* (Figure 6q–t). High relative amounts of the LM6 epitope in some fern samples (e.g. *Asplenium ceterach* and *Asplenium ruta-muraria*) are caused by a high vascular tissue to total tissue ratio. In contrast to the leptosporangiate ferns, where LM6 was largely restricted to vascular and epidermal tissue, it was immunodetected in the majority of tissues in *Equisetum* (see [37]), explaining the relative high amounts of the LM6 epitope in both extractions in the glycan array analysis. While we did not detect the LM6 epitope in *Huperzia*, we observed a similar distribution pattern in *Selaginella* as observed in leptosporangiate ferns; a detailed study focussing on cell wall composition of lycophytes is needed to confirm the absence of this epitope in homosporous lycophytes.

**Figure 7:**
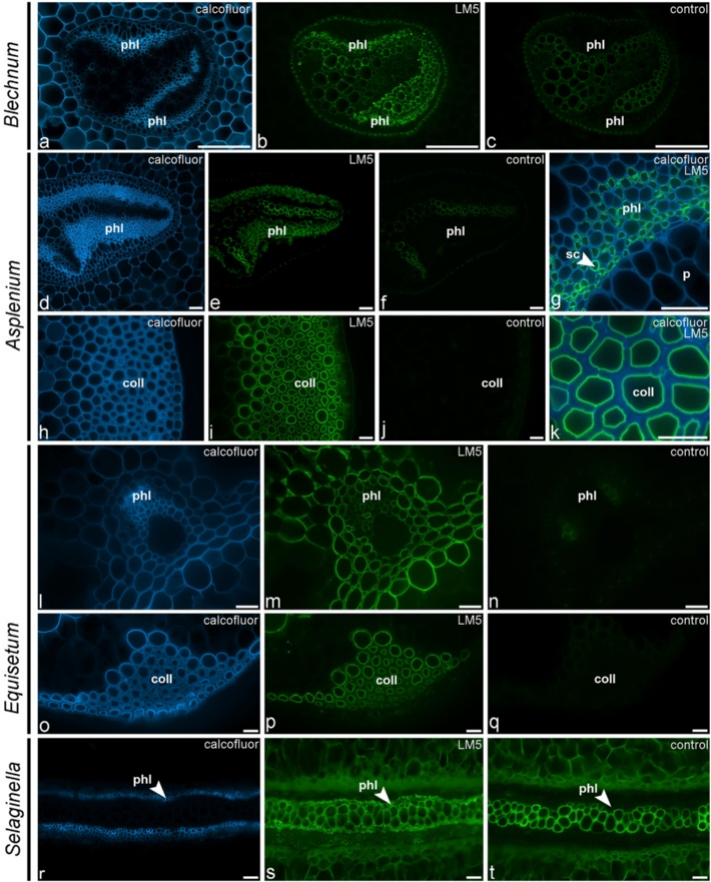
**Indirect immunofluorescence detection of the galactan (LM5) epitope in transverse sections of fern petioles and lycophyte stems.** Calcofluor White fluorescence **(a, d, g, h, k, l, o, r)** shows the full extent of cell walls. **(a–c)** Abundance of the LM5 epitope in cell walls of phloem sieve cells in the vascular bundle of *Blechnum brasiliense*. **(d–g)** Similar distribution pattern of LM5 in *Asplenium compressum*. A high magnification **(g)** of a vascular bundle shows that LM5-binding is restricted to cell walls of phloem sieve cells (sc). **(h–k)** Binding of LM5 to the innermost cell wall layers of collenchymatous tissue in *Asplenium rutifolium*. **(l–q)** LM5 binding to most tissues in *Equisetum arvense*, including the cell walls of the vascular bundle and surrounding parenchyma **(l–n)** as well as to the inner cell wall layer of the collenchymatous strengthening tissue **(o–q)**. **(r–t)** LM5 binding to phloem in the lycophyte *Selaginella grandis*. Abbreviations: phl, phloem; sc, sieve cell; coll, collenchymatous tissue. No primary antibody controls are provided **(c, f, j, n, q, t)**. Scale bars: 40 μm.

The galactan LM5 epitope was detected in the NaOH extract of most leptosporangiate ferns and lycophytes (Figures 2 and 4). Remarkably, LM5 was found in high amounts in the CDTA cell wall extractions for all *Equisetum*-species and *Danaea*, suggesting that they might have distinct cell wall architectures compared to all other ferns and lycophytes studied where NaOH was required to extract the LM5 epitope (Figure 3). These results correlate with phylogenetic studies presenting evidence that the marattioid ferns are nearest (extant) relatives of horsetails [43]. Similarly to LM6, a high abundance of the LM5 epitope was found in a sample containing isolated vascular bundles of *Asplenium elliottii* (Figure 2). This was supported by IF as we immunodetected galactan in the walls of phloem sieve cells in all leptosporangiate ferns as shown for *Blechnum* and *Asplenium* (Figure 7a–g). Additionally, LM5 bound to the inner cell wall layers of collenchymatous tissues (e.g., *Asplenium theciferum*, *Asplenium loxoscaphoides*, *Asplenium compressum*) as shown for *A. theciferum* in Figure 6h–k. In *Equisetum* and *Angiopteris*, galactan ― in accordance with the LM6 arabinan epitope ― was detected in most tissues as shown for *Equisetum* in Figure 7l–q. In the lycophyte *Huperzia*, strong binding of LM5 to cortical parenchyma and weak binding to phloem cells was observed (data not shown). In *Selaginella*, the LM5 epitope was detected in the phloem tissue (Figure 7r–t). Pectate lyase treatment generally resulted in stronger binding of LM5, but unmasking was not observed in tissues where no LM5 epitope was detected in untreated sections. It has been suggested that the occurrence of RG-I and its structural variants can be related to mechanical properties of cells or developing organs [42,44-46]. Although the structure-function relationships of galactan-rich pectins are still poorly understood, the literature [35,47] suggests that these polymers might play an important structural and/or regulatory role in mechanically stressed cell walls. It is of interest to note that we immunodetected LM5-epitopes in the walls of sieve cells and collenchymatous cells, cell types which undergo extensive elongation during differentiation. The identification of the LM5 epitope in distinct cell and tissue locations from those observed for the LM6 epitope indicates that they are binding to specific polysaccharides and how these relate to the rhamnogalacturonan-I structures of angiosperms remains to be determined.

### Xyloglucan epitopes associated with phloem tissues and, after unmasking, primary cell walls

Xyloglucans have a backbone of (1 → 4)-β-d-glucan units, some of which are substituted with short side chains [31,48]. The structure of xyloglucan can be highly complex, and often shows variation in different taxonomic orders in different plant groups [49,50]. LM15, binding to the XXXG-motif of xyloglucan (although it also binds to tobacco xyloglucan with a XXGG motif [24]), and CCRC-M1, binding to fucosylated xyloglucan were employed in this study. Xyloglucans are the most abundant hemicelluloses in primary walls of seed plants, except for grasses and other commelinid monocotyledons except for palms, where (glucurono)arabinoxylans are the major hemicelluloses [12,21,48]. They have also been detected in primary cell walls of bryophytes [9,10,51,52], lycophytes, ferns and gymnosperms [9,10,19,50] and immunolabelling experiments indicated their presence in some charophycean green algae [30,40,53].

**Figure 8:**
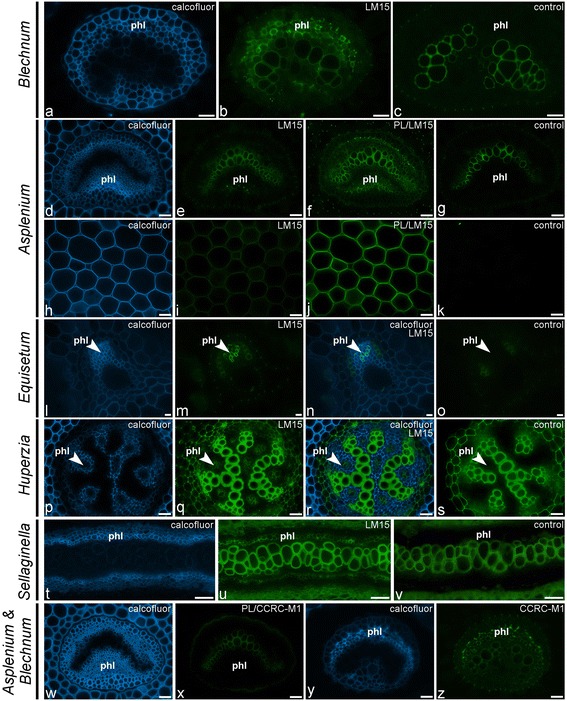
**Indirect immunofluorescence detection of xyloglucan (LM15, CCRC-M1) epitopes in transverse sections of fern petioles and lycophyte stems.** Calcofluor White fluorescence **(a, d, h, l, n, p, r, t, w, y)** shows the full extent of cell walls. **(a–c)** Binding of LM15 to phloem cell walls of *Blechnum brasiliense*. **(d–k)** Pectate lyase treatment (PL) unmasks LM15-epitopes in phloem cell walls of *Asplenium rutifolium* **(d–g)** and in primary cell walls of cortical parenchyma of *Asplenium elliottii* **(h–k)**. **(l–o)** LM15 binds to phloem tissue of *Equisetum ramosissimum*. **(p–v)** Similar distribution patterns are found in the lycophytes *Huperzia squarrosum* **(p–s)** and *Selaginella grandis* **(t–v)**. **(w–z)** While the CCRC-M1 epitope is not detected in *Asplenium rutifolium* **(w, x)**, even after pectate lyase pre-treatment (PL), it is localized in the phloem of *Blechnum brasiliense* **(y, z)**. No primary antibody controls are provided **(c, g, k, o, s, v)**. Abbreviations: phl: phloem. Scale bars: 40 μm.

Glycan microarray analysis indicated the presence of the LM15 xyloglucan epitope in the NaOH-extracts of most of the fern and lycophyte species studied (Figures 2 and 4). In our analysis of isolated tissues a relatively high amount of LM15 was detected in isolated vascular bundles in both the CDTA- and NaOH-extracts (Figure 2). IF confirmed this as we observed binding of LM15 to phloem cell walls (Figure 8a–g). After pectate lyase treatment the latter binding signal was stronger and, in addition, binding to cortical parenchyma cell walls was also observed (Figure 8h–k). This shows that, as in angiosperms, LM15 mAb binding often requires enzymatic removal of HG [24]. The LM15 epitope was restricted to phloem cell walls (Figure 8l–o) and guard cell walls in *Equisetum* [41] and was detected in the phloem and cortex of the lycophytes *Huperzia* and *Selaginella* (Figure 8p–v). In *Psilotum*, LM15 bound to the inner zones of the cortex, as well as to the phloem. The immunodetection of LM15 in the phloem of all early tracheophytes suggests that xyloglucan ― or its structural elaboration ― may have played an important role in the evolution of phloem, and that its incorporation within the phloem walls has been conserved during the evolution of land plants, as xyloglucan has also been immunolocalised in angiosperm phloem [24].

The CCRC-M1 fucosylated xyloglucan epitope was detected in the NaOH-extracts of most leptosporangiate and eusporangiate ferns studied, but, with the exception of a very weak signal in two species, not found in the Aspleniaceae (Figures 2 and 4). Within the lycophytes, the CCRC-M1 epitope was only detected in the heterosporous *Selaginella*. IF confirmed these observations as CCRC-M1 was not detected in Aspleniaceae (Figure 8w, x) and widely immunodetected in other leptosporangiate ferns such as *Blechnum* (Figure 8y, z), treated or untreated with pectate lyase. In non-asplenioid leptosporangiate ferns, CCRC-M1 bound to phloem cell walls (Figure 8y-z), which further suggests that xyloglucan might have been important for the evolution of phloem tissues. The absence of this epitope in most asplenioid ferns indicates that its abundance or detectability is variable at family level. Although high relative amounts (compared to LM15) of the CCRC-M1 epitope were detected in our glycan array analysis, IF only revealed weak binding, even after pectate lyase pretreatment, suggesting that CCRC-M1 epitopes might be masked by other polymers than HG or are soluble and lost during antibody-incubation procedures. As the epitope was found in two out of 36 species belonging to Aspleniaceae, it is probably only present in very low amounts or in a configuration that hinders epitope access or alters extractability.

### Xylan epitopes are associated with secondary cell walls but also display some distinct distribution patterns

The mAbs LM10 and LM11 both recognise unsubstituted (1 → 4)-β-xylan, but LM11 can also bind to substituted arabinoxylans [54]. Xylans are the major cellulose-linking polysaccharides in secondary cell walls of higher plants [12,48] and are the major non-cellulosic polysaccharides in primary cell walls of commelinid monocots [12,48]. In ferns, xylans have been reported to occur in secondary cell walls [55,56]. Evidence for the presence of xylans in charophycean green algae, chlorophytes, and red algae has also been published (e.g. [53,57]). In angiosperms, LM10 and LM11 bind strongly to secondary cell walls, and pectate lyase pretreatment unmasked xylan epitopes in parenchyma, including distinct regions of collenchyma cell wall thickenings [58].

**Figure 9:**
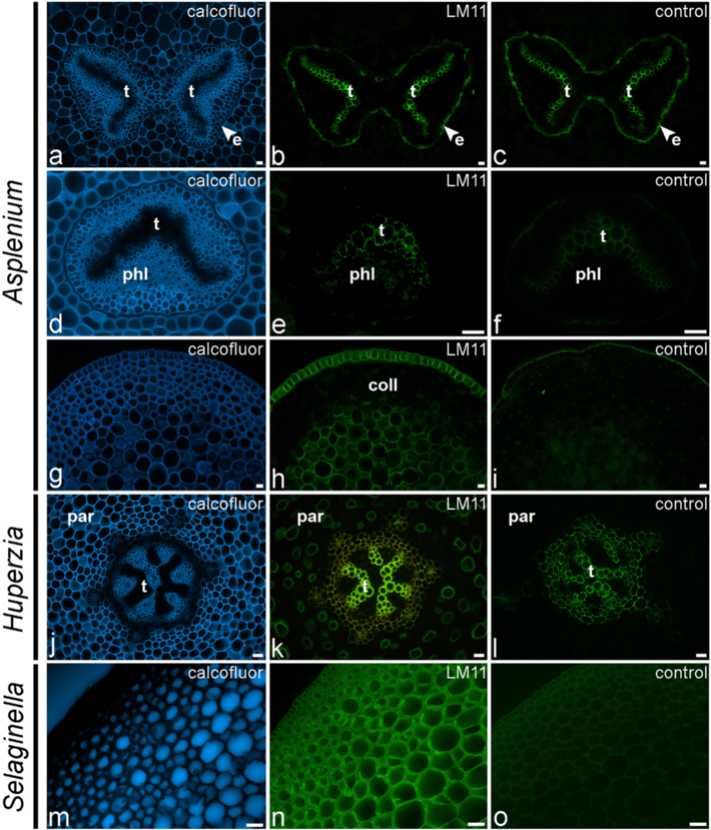
**Indirect immunofluorescence detection of the xylan (LM11) epitope in transverse sections of fern petioles and lycophyte stems.** Calcofluor White fluorescence **(a, d, g, j, m)** shows the full extent of cell walls. **(a–c)** Autofluorescence of tracheids (t) obscures observation of LM11 binding in *Asplenium polyodon*. Note also the autofluorescence of the suberin lamellae of the endodermis **(e)**. **(d–f)** LM11 binding to tracheid cell walls and phloem cell walls of *Asplenium loxoscaphoides*. **(g–i)** Abundance of the LM11 epitope in epidermal cell walls and cortical parenchyma of *Asplenium rutifolium*. Note that the epitope is not detected in the collenchymatous tissue (coll). **(j–l)** Binding of LM11 to cell walls of scattered cortical parenchyma cells in the lycophyte *Huperzia squarrosum*. **(m–o)** In the lycophyte *Selaginella grandis*, the LM11 epitope is detected in all cortical parenchyma cell walls. Abbreviations: t, tracheids; e, epidermis; phl, phloem; coll, collenchymatous tissue. No primary antibody controls are provided **(c, f, i, l, o)**. Scale bars: 40 μm.

Glycan microarray analysis only detected the LM11 epitope in the NaOH-extract of a small number of leptosporangiate ferns and heterosporous lycophytes and the LM10 epitope in *Diplazium* (Figure 4). We did not detect these epitopes in isolated vascular bundles (Figure 2), suggesting that the total amount of xylan epitopes might be relatively low compared to that of other epitopes, or that the epitope was not extracted in sufficient amount to be detected. Interestingly, none of these epitopes have been found in a sample of isolated sclerenchyma (Figure 2), suggesting that, in some ferns, xylans might not be the major hemicellulosic polysaccharides in sclerified tissues, unless they occur as a structural variant which cannot be recognised by either LM10 or LM11. With few exceptions, LM11 gave much the same IF results, binding to tracheid cell walls (Figure 9a–f). However, in several cases autofluorescence did not allow clear observation of antibody binding as shown for *A. polyodon* in Figure 9a–c. IF did not provide clear evidence of LM10 binding to cell walls of any fern or lycophyte studied; weaker binding compared to that of LM11 (as reported previously [58]) could easily have been obscured by autofluorescence. In only a few cases LM11 weakly bound to phloem parenchyma cells (Figure 9d–f) and to cortical and epidermal primary cell walls, but not to collenchymatous cell walls, as shown for *A. rutifolium* (Figure 9g–i). Interestingly in other petioles of the same *A. rutifolium* plant we did not observe LM11 binding to the cortical parenchyma and epidermis (data not shown). In *Huperzia squarrosum*, LM11 bound to xylem tracheids and scattered cells of the cortex parenchyma (Figure 9j–l), whereas in the heterosporous lycophyte *Selaginella* it strongly bound to all cortical parenchyma cell walls (Figure 9m–o). The unusual binding pattern in *Huperzia* suggests that sclerification of cortical parenchyma might not occur simultaneously in all parenchyma cells. We specifically immunodetected the LM11 epitope in *Equisetum* guard cell walls (data not shown). It is of interest that the same epitope was also found in guard cells of *Psilotum* [56], suggesting that they might fulfil a structural role in stomata of early tracheophytes.

### Mannan-epitopes are associated with sclerified secondary cell walls and are unmasked in primary cell walls after pectate lyase treatment

Mannans are mannose-rich complex heteroglycans that can occur as storage polymers, particularly in the leguminosae [59], or fulfil structural functions [48]. In this study we used the LM21 antibody which is known to bind to heteromannans including glucomannan and galactomannan [60]. Mannans appear to be very abundant in the primary cell walls of the earliest land plants such as bryophytes, lycophytes, and early-diverging ferns, and less abundant in those of leptosporangiate ferns, gymnosperms and angiosperms [9,10,60-63]. A mannan-rich primary cell wall type was proposed for ferns, based on analysis of fern laminae [23]. Mannans have also been detected in algal species [30], including some of which completely lack cellulose in their cell walls [64,65]. In angiosperms, mannans (mainly glucomannans and galactoglucomannans) are usually found at low levels in secondary cell walls [12,66,67], whereas they are the major hemicelluloses in secondary cell walls of gymnosperms [12,66]. Glucomannans, in particular, give extremely tough physical characteristics to some cell walls [31].

**Figure 10:**
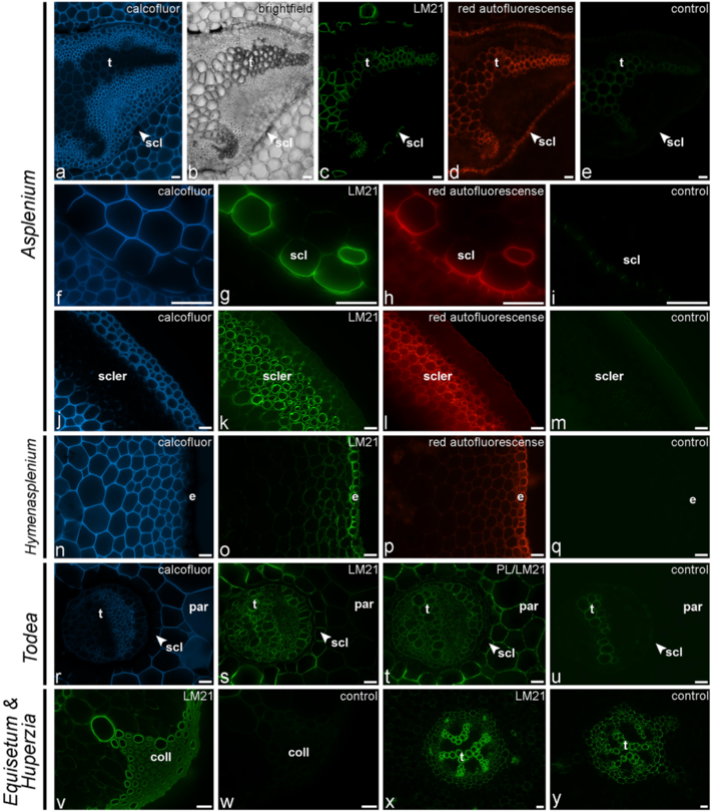
**Indirect immunofluorescence detection of mannan (LM21) epitopes in transverse sections of fern petioles and lycophyte stems.** Calcofluor White fluorescence **(a, f, j, n, r)** and bright-field **(b)** showing the full extent of cell walls. **(a–i)** Localisation of the LM21 epitope in and around the vascular bundle of *Asplenium elliottii*. The epitope is detected in the cell walls of tracheids **(t)** and sclereids (scl) that surround the vascular bundle. A high magnification shows detection of the LM21 epitope in the inner cell wall layer of sclereids **(f–i)**. Note red autofluorescence of sclereid cell walls **(d, h)**. **(j–m)** LM21 binding to the innermost cell wall layer of sclerenchyma hypodermal cells (hyp) and the sclereids of an *Asplenium elliottii* petiole. Note red autofluorescence of sclereid cell walls **(l)**. **(n–q)** Immunodetection of LM21 in sclerified epidermal cell walls and subepidermal tissue of *Hymenasplenium obscurum*. Note red autofluorescence of sclerified epidermis and subepidermal tissue **(p)**. **(r–u)** Unmasking of the LM21 epitope in primary cell walls of the cortical parenchyma of *Todea sp.* after pectate lyase treatment (PL). Note that cell walls of the vascular bundle and surrounding sclereids (scl) are labeled prior to pectate lyase treatment. **(v, w)** Detection of the LM21 epitope in the collenchymatous strengthening tissue of *Equisetum ramossisimum*. **(x, y)** Strong binding of LM21 to tracheids and weak binding to cortical parenchyma in the lycophyte *Huperzia squarrosum*. Abbreviations: scl: sclerenchyma; phl: phloem; ep: epidermis. Abbreviations: t, tracheids; scl, sclereids; scler, sclerified tissue; e, epidermis; par, parenchyma, coll, collenchymatous tissue. No primary antibody controls are provided **(e, i, m, q, u, w, y)**. Scale bars: 40 μm.

Glycan microarray analysis detected mannans in both NaOH- and CDTA-extracts of ferns and lycophytes (Figures 2, 3 and 4). High amounts of the mannan-epitope were found in species with relative high amounts of sclerified tissues. In *A. elliottii* the highest amounts of mannan-epitopes were detected in the isolated hypodermal sclerenchyma (NaOH-extract), petiole base tissue and vascular bundles (CDTA-extract) (Figure 2). IF showed that throughout all ferns and lycophytes studied, the LM21-epitope is strongly associated with (sclerified) secondary cell walls (Figure 9). LM21 strongly bound to the inner cell wall layer of sclerified epidermal and hypodermal cell walls, scattered sclerified cortex parenchyma cells as well as to xylem tracheids and sclereids surrounding the vascular bundles (Figure 10a–q). As the LM21 epitope was restricted to the inner cell wall layers of secondary cell walls, it is possible that these epitopes might be masked as a result of the incorporation of (autofluorescent) phenolic compounds. In some cases we observed weak binding of LM21 to cortical parenchyma and the vascular bundle. After pectate lyase pretreatment LM21 bound to primary cell walls in most fern species (Figure 10r–u). LM21 was also immunodetected in the strengthening tissue of *Equisetum* (Figure 10v, w) and in tracheids of *Huperzia* (Figure 10x, y). These results show that heteromannans are present in fern primary cell walls and that they are also implicated in the sclerification process. The walls of these mechanical tissues are typically impregnated with yellow-brown phenolic pigments. The absence of the LM11-epitope in sclerenchyma suggests that, as in gymnosperms, heteromannans might be the major hemicelluloses in secondary cell walls. The presence and abundance of hemicelluloses in fern cell walls may be different from what is generally found in angiosperm cell walls. While primary cell walls of (dicotyledonous) flowering plants are generally xyloglucan-rich [31], we detected mannan and xyloglucan epitopes in fern primary cell walls. Our results also suggest that mannans, as in gymnosperm secondary walls [12], might be the dominant hemicellulose in most fern secondary walls. These findings are in agreement with earlier reports stating that fern cell walls, either primary or secondary, are mannan-rich [9,10,23]. However, results obtained through IF by themselves do not establish the major hemicelluloses and therefore, detailed investigations of fractioned cell wall material from different fern tissues are needed to determine the relative abundance and structural variation of hemicelluloses. This might increase insights into the functional redundancy or interchangeability of sets of hemicellulosic polymers pertaining to cell wall properties.

### Mixed-linkage glucan is detected in cell walls of fully elongated cells

**Figure 11:**
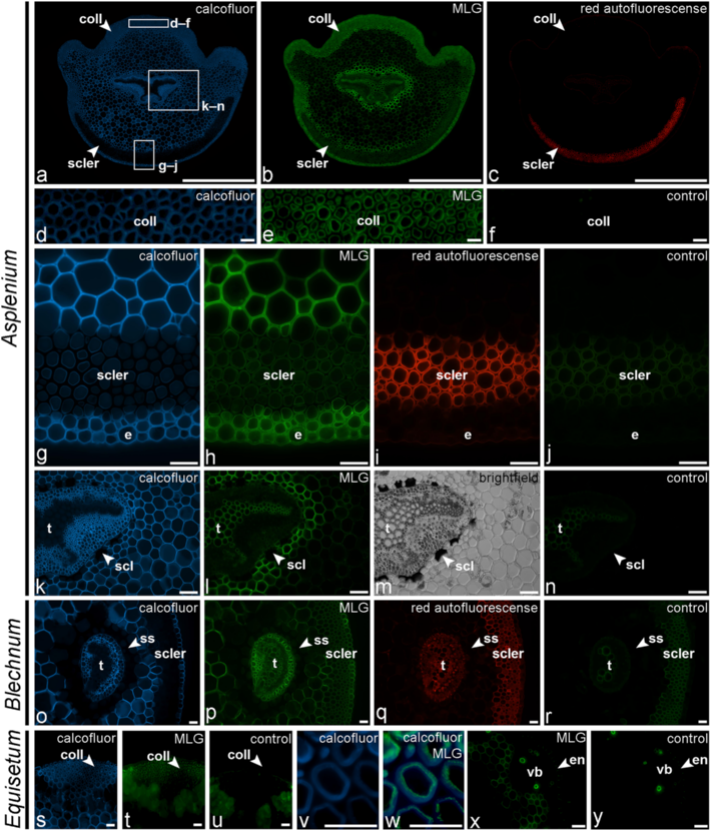
**Indirect immunofluorescence detection of the mixed-linkage glucan epitope (BioSupplies 400–3) in transverse sections of fern petioles and lycophyte stems.** Calcofluor White fluorescence **(a, d, g, k, o, s, v, w)** and bright-field **(m)** showing the full extent of cell walls. **(a–n)** Localisation of the MLG epitope in *Asplenium elliottii*. The epitope is detected in collenchymatous **(d–f)** and sclerenchymatous **(g–j)** mechanical tissues as well as in epidermal cell walls. Parenchyma shows differential labelling intensities with stronger labelling of parenchyma walls surrounding mechanical tissues. Increased labelling is observed in parenchyma tissues bordering the hypodermal sclerenchyma **(g–j)** and the zone with sclereids surrounding the vascular bundle **(k–n)**. Weak labelling is observed in the phloem tissue. **(o–r)** The anti-MLG antibody binds to parenchymatous cell walls in *Blechnum brasiliense*. Cell walls of the sclerenchyma sheath (ss) and subepidermal sclerenchyma (scler) are not labelled. **(s–w)** Detection of the MLG epitope in the collenchymatous strengthening tissue (coll) of *Equisetum arvense*. Higher magnification **(v, w)** shows that the epitope is restricted to secondary cell walls. **(x, y)** Labelling of a continuous ring of parenchyma tissue located between the central cavity and vascular bundles. Abbreviations: coll, collenchymatous tissue; scler, sclerenchyma; e, epidermis; scl: sclereïds; t, tracheids; ss, sclerenchyma sheath; vb: vascular bundle. No primary antibody controls are provided **(f, j, n, r, u, y)**. Scale bars: **a**–**c**, 1 mm; **d**–**w**, 40 μm.

(1,3;1,4)-β-d-glucans, also known as mixed-linkage glucans (MLGs), are found in species belonging to the angiosperm Poales order [12] but have also been detected in distantly related *Equisetum*-species [19,20]. Recently, the BS-400-3 MLG epitope was detected in the lycophyte *Selaginella moellendorfii* [68] and it was shown that its detection was abolished after lichenase treatment, indicating that MLGs are more common in vascular plants than previously assumed. Based on thin-layer chromatography of lichenase digests, Xue and Fry [69] explored the occurrence of MLG in a selection of lycophytes and ferns s.l. (including *Equisetum*). They did not find evidence for the presence of MLG in the ten leptosporangiate ferns species they have studied. Our glycan microarray analysis, however, revealed variable amounts of the MLG-epitope in the NaOH extracts of ferns and lycophytes, from nearly undetectable to high relative values of over 90 (Figure 4). Two of the ten leptosporangiate ferns investigated by Xue and Fry [69], *Todea barbara* and *Trichomanes speciosum*, were also included in the present study and in which no or very low relative amounts of the MLG epitope were detected. In *Equisetum*, a higher abundance of MLG was found in species belonging to the subgenus *Equisetum* compared to representatives of the subgenus *Hippochaete* [69]. Our results show the same trend as considerably lower relative levels of the MLG epitope were detected in *E. hyemale* and *E. ramosissimum*, which both belong to the *Hippochaete* subgenus. IF showed that the MLG antibody binds to different tissues in the leptosporangiate fern *Asplenium elliotii* (Figure 11a–n). While the thickened walls of the collenchymatous mechanical tissue were strongly labelled, the epitope was not detected in the abaxial sclerenchyma tissue (Figure 11a–j). This, however does not imply that MLG is absent in the latter tissue, as the epitope could be masked by phenolic impregnation of the sclerenchyma walls. The MLG-epitope was also detected in the epidermis (Figure 11g–j). Parenchymatous tissues were weakly labelled except for cell walls of the cell layers surrounding mechanical tissues, where increased binding signals were detected (Figure 11k–n). Labelling in the vascular bundle was restricted to the pericycle (Figure 11k–n). In *Blechnum brasiliense* both cortex parenchyma and pericycle were labelled, while cell walls of the sclerenchyma sheath and subepidermal sclerenchyma were not (Figure 11o–r). In *Equisetum*, we immunodetected the MLG epitope in the thickened cell walls of the strengthening tissue and the epidermis (Figure 11s–w), as well as in cell walls of a continuous ring of parenchymatous tissue located between the central cavity and the vascular bundles (Figure 11x, y). In all cases binding of the anti-MLG antibody was abolished after lichenase treatment (data not shown). It is of interest that species including *Todea barbara*, *Osmunda vauchellii*, *Trichomanes speciosum*, *Adiantum capillus-veneris* and *Asplenium hemionites*, for which we did not detect MLG-epitopes in the glycan array, contain high proportions of sclerenchyma and have very stiff petioles. Species with collenchymatous mechanical tissues, on the other hand, including *Equisetum*, *Asplenium nidus* and *Asplenium tenerum*, were found to contain high relative amounts of the MLG-epitope. Moreover, the localisation of the epitope within or close to areas where sclerification may occur suggests that MLGs may (1) indirectly or directly mediate cell wall mechanical properties, especially in plant organs with immature sclerenchyma tissues and/or (2) play a role in the sclerification process. This is in agreement with the results of the glycan array analysis of different organ and tissue samples of *Asplenium elliottii* (Figure 2). In the petiole, the highest relative amount of MLG epitopes was found in the middle section of the petiole, which is characterised by abaxial sclerified and adaxial collenchymatous mechanical tissues (Figure 11a–c). In addition, it was found that, in *Equisetum*, MLGs were more abundant in mature non-growing stem base regions than in younger tissues [19]. Accumulation of MLG in mature, not elongating cell walls is, however, not restricted to ferns s.l. as the MLG-epitope has also been detected in grass fibers and xylem vessels [70-72], which contain thick, lignified, secondary cell walls that provide mechanical support. Further analytical investigations are required to confirm the presence and determine the composition of MLG in ferns. Spatio-temporal distribution patterns in relation to growth and development are likely to provide a deeper insight into the functional role of MLG in fern cell walls.

## Conclusions

The molecular probes used in this analysis have been developed to study angiosperm taxa but are clearly applicable to an analysis of fern cell walls indicating that at least some of the cell wall structures present in angiosperm cell wall polysaccharides are conserved. As cell walls are highly dynamic with their chemical composition depending on developmental and environmental cues, it is not surprising that the glycan array data set displayed variation in glycan epitope-distribution among ferns but also within Aspleniaceae. However, IF of selected species enabled identification of glycan epitopes that were recurrently detected in cell walls of specific tissues and tissue types, indicating that changes in cell wall architecture, including structural elaboration of specific polymers, are linked to the emergence and/or differentiation of specialised tissues and cell types in land plants. In all species studied we detected xyloglucan LM15 and xylan LM11 epitopes in phloem and xylem tracheid walls, respectively. The RG-I-related LM5- (galactan) and LM6-epitopes (arabinan) were found in cell walls of specific cell-types in vascular bundles in all leptosporangiate ferns. The LM21 heteromannan epitope was generally detected in cell walls of mechanical tissues that are impregnated with yellowish-brown pigments, but was also found in primary cell walls, typically after pectate lyase pretreatments. To the best of our knowledge we provided the first evidence of MLG occurrence in leptosporangiate ferns, in which we detected the highest abundance of the BS-400-3 MLG epitope in petioles with immature but fully elongated sclerenchyma tissues.

Our results also provided further evidence indicating the distinctive nature of *Equisetum* cell walls and indicated that only low amounts of pectic HG epitopes were detected in CDTA extracts of lycophytes. We showed that while the fucosylated xyloglucan epitope CCRC-M1, except for low amounts in two species, was not detected in the fern family Aspleniaceae, high relative amounts were found in other leptosporangiate fern species. In the same way, the CCRC-M1 epitope was detected in the heterosporous *Selaginella* species but not in the homosporous lycophyte *Huperzia*. These few examples further emphasize the complexity cell wall diversity as cell wall polysaccharides not only vary in their fine-structural details but also may display intricate spatio-temporal and phylogenetic distribution patterns.

## Implications for screening cell wall diversity

The variation in cell wall composition at lower taxonomic levels raises the question which criteria should be used to select species that are representative of specific plant lineages.

An approach that combines high-throughput screening with detailed analysis of selected species, as adopted here and discussed elsewhere [18], partly solves this problem, allowing investigation of large numbers of samples. However, while covering as much diversity as possible is important, one also needs to take into account other sources of variation. The position of a cell wall in the plant, organ or tissue at any given developmental stage, as well as the environment in which the plant occurs all have an impact on cell wall architecture. The extent of and susceptibility to variation may be substantial as, in contrast to most animals, plants have a sedentary lifestyle. Hence, they display plastic phenotypes, resulting in often dramatic differences between conspecifics or even among organs produced by the same plant as we highlighted in this study. IF is one of the best methods to unravel such complexity allowing fine mapping of cell wall composition at different levels of organisation, from organs to cell wall microdomains at any given developmental stage. Several of our results emphasize the merits of including antibody-based techniques in cell wall comparative studies. We found that differences in epitope abundance are frequently related to the relative proportions of specific tissues with distinct cell wall composition. In addition, some components may have spatially restricted distributions and may be present in amounts that are below the detection limit of some analytical techniques. We also demonstrated variation in cell wall composition within heterogeneous tissues as well as in individual cell walls, information that is often lost during fractionation of plant cell walls for polysaccharide isolation. However, to fully understand cell wall diversity and evolution a multi-modal/-scale approach is necessary, and while *in situ* labelling experiments clearly have some advantages, they should ideally be complemented with biochemical analysis of fractionated cell walls to provide conclusive information on polymer structure and presence.

## Methods

### Plant material

Seventy six fern species from 20 families and 4 species of lycophytes growing in the fern collection at the Ghent University Botanical Garden were sampled for material that was used in our glycan microarray analysis with 15 cell wall directed molecular probes (Figure 1, Supplementary information Additional file 1). In order to explore infrageneric variation a larger number of species belonging to the family Aspleniaceae (36 species) were collected.

For glycan microarray analysis of organs and tissues of *Asplenium elliottii* (root, rhizome, petiole, lamina, vascular bundles, sclerenchyma and cortex parenchyma parenchyma) we sampled base- mid and top section of petioles and manually isolated tissues from the petioles with single edge razor blades. First petioles were dissected longitudinally and the vascular bundles were pulled out. Second, the soft epidermis and cortical parenchyma tissues were removed by scraping them off the hard and brown-coloured sclerenchyma tissue. In a second glycan microarray analysis we sampled petioles bases only (or stems in the case of *Huperzia*, *Selaginella*, *Psilotum* and *Equisetum*) of mature plants as these organs contain ground, vascular and mechanical tissues, and their rigidity allows easy vibratome sectioning. We primarily collected material from plants cultivated in plant beds or large containers as small container size may inhibit plant growth and, hence, affect differentiation of mechanical tissues, which may influence overall cell wall composition. Material was also fixed for sectioning (see further below).

### Alcohol Insoluble Residue (AIR)

Material was suspended in liquid nitrogen and homogenized to a fine powder using a mortar and pestle. Five volumes of aqueous 70% ethanol were added and the suspension samples incubated for 1 h at 4°C on a rotator, centrifuged (10,000 × g for 10 minutes) and the supernatant discarded. This procedure was repeated 5 times, before a final wash with acetone for 2 min. The AIR was then air dried.

### Glycan microarray analysis

Glycan microarray profiling was performed on AIR samples as previously described [73]. Pectins, and polymers associated with pectins, were extracted by vortexing three sample replicates, consisting of 5 mg of AIR each, with 150 μl 50 mM CDTA (1,2-Diaminocyclohexane-N,N,N’,N’-tetraacetic acid), pH 7.5 in a TissueLyser (Qiagen MM 200) at 6 shakes s-1 for 2h. After centrifuging at 12,000 × g, supernatants (CDTA-extracts) were removed and stored at 4°C. After washing the pellets with de-ionized water, they were incubated with 150 μl of 4M NaOH with 0.1% v/v NaBH4 in the TissueLyser at 6 shakes s-1 for 2h to extract hemicelluloses. After centrifuging at 12,000 × g, supernatants (NaOH-extracts) were removed again and kept at 4°C. Extracts were spotted onto nitrocellulose membranes using a Sprint microarrayer (ArrayJet, Roslin, Scotland, UK). The original extracts, plus two dilutions (1:2 and 1:5) were printed in triplicate (printing replicates). Arrays were air-dried and probed with mAbs. Briefly, after blocking with 5% w/v milk protein in PBS, arrays were incubated in primary mAbs (1:10 dilution for CCRC-M1 and LM mAbs, 1:100 for BS-400-3 mAb) for 2h at room temperature. After washing, arrays were probed with secondary antibodies conjugated to alkaline phosphatase for 1.5 h before washing and developing in a BCIP/NBT (5-bromo-4-chloro-3-indolyphosphate/nitro-blue tetrazolium chloride) substrate. Arrays were then scanned and uploaded into ImaGene 6.0 microarray analysis software (BioDiscovery, http://www.biodiscovery.com). Mean spot signals (spot signals corresponding to just one dilution value on the array were used) from the three independent experiments are presented as a heatmap with the values normalized to the highest value of the entire dataset (set to equal 100). A cut off of 5% of the highest mean signal value was imposed and values below this are represented as 0.

### Indirect immunofluorescence analysis of cell wall epitopes

Materials were fixed in 4% (w/v) paraformaldehyde in 50 mM PIPES (1,4-piperazinediethanesulfonic acid), 5 mM MgSO_4_, and 5 mM EGTA (ethylene glycol tetraacetic acid), pH 6.9. After washing in phosphate-buffered saline (PBS), transverse 40–60 μm thick sections were cut from unembedded material using a Microm HM650V vibration microtome (Thermo Fisher Scientific, Walldorf, Germany).

For indirect immunolabelling, sections were incubated in 5% w/v milk protein in PBS (MP/PBS) for 5 min to block non-specific binding sites. Sections were then incubated with primary monoclonal antibodies diluted in MP/PBS (LM19, LM20, LM5, LM6, LM15, LM10, LM11, LM21, www.plantprobes.net, diluted 1:10; CCRC-M1, http://www.ccrc.uga.edu/~carbosource/CSS_home.html, diluted 1:5; and BS-400-2, http://www.biosupplies.com.au/, diluted 1:25) for 1 h (for specificity of probes see Table 1). Negative control staining was carried out by omitting the primary antibodies. After washing with several changes of PBS, sections were incubated with anti-mouse-IgG (CCRC-M1 and BS-400-3) or anti-rat-IgG (remaining antibodies), both linked to fluorescein isothiocyanate (FITC; Sigma) and diluted 1:100 in 5% w/v MP/PBS for 1 h. Some sections were pretreated with either 10 μg/mL Pectate lyase (from *Cellvibrio japonicus*, Megazyme, Bray, Ireland) for 2h at room temperature in 50 mM N-cyclohexyl-3-aminopropane sulfonic acid (CAPS), 2 mM CaCl_2_ buffer at pH 10 as described previously [24] or 0.2 mg/mL lichenase (mixed-linkage glucan specific enzyme, from *Bacillus subtilus*, Megazyme, Bray, Ireland) in sodium phosphate buffer (pH 6) for 2h at 37°C. Counterstaining was performed with Calcofluor White M2R fluorochrome (fluorescent brightener 28; Sigma; 0.25 μg mL^−1^ in dH_2_O). Subsequently, all sections were washed in PBS three times before mounting in a glycerol-based anti-fade solution (Citifluor AF2, Citifluor Ltd., UK). Immunofluorescence was observed with an epifluorescence microscope (Olympus BX-61) equipped with the following filter sets: 350/450 nm (ex/em) for visualizing calcofluor white stained cell walls; 490/520 nm (ex/em) for green emission of the FITC fluorochrome, and, 541/572 nm (ex/em) for imaging red autofluorescence of brown-coloured sclerified cell walls (referred to as ‘red autofluorescence’). Images were captured with a Hamamatsu ORCA285 camera and prepared with Volocity software.

**Table 1:** **List of monoclonal antibodies used in this study**

	**mAb**	**Specificity**	**References**
Pectic HG/related	JIM5	partially or demethyl-esterified HG	[26]
	JIM7	partially methyl-esterified HG	[26]
	LM19	partially or demethyl-esterified HG	[27]
	LM20	methyl-esterified HG	[27]
	LM7	non-blockwise partially methyl-esterified HG	[25,27]
RG-I/related	LM5	(1 → 4)-β-galactan	[76]
	LM6	(1 → 5)-α-arabinan	[34]
	LM8	xylogalacturonan	[77]
	LM9	feruloylated (1 → 4)-β-D-galactan	[78]
Hemicelluloses/XG	CCRC-M1	fucsoylated xyloglucan	[79]
	LM15	XXXG-motif of xyloglucan	[24]
Hemicelluloses/xylan	LM10	(1 → 4)-β-d-xylan	[54]
	LM11	(1 → 4)-β-d-xylan / arabinoxylan	[54]
Hemicelluloses/mannan	LM21	heteromannan	[60]
Hemicelluloses/mixed-linkage glucan	BS-400-3	(1 → 3, 1 → 4)-β-d-glucan	[80]

HG = homogalacturonan, RG-I = rhamnogalacturonan-I, XG = xyloglucan, MLG = mixed-linkage glucan.

## Additional file

Additional file 1:
**List of studied material with voucher information.** BGUG: Collection of the Botanical Garden University of Ghent.
