# Supplementary material for: Antibody-based screening of cell wall matrix glycans in ferns reveals taxon, tissue and cell-type specific distribution patterns
Source: BMC Plant Biol. 2015 Feb 18;15:56. doi: 10.1186/s12870-014-0362-8 (PMC4351822; doi:10.1186/s12870-014-0362-8)
Supplement: Additional file 1: — List of studied material with voucher information. BGUG: Collection of the Botanical Garden University of Ghent. [file 12870_2014_362_MOESM1_ESM.pdf]

Appendix 1: List of studied material with voucher information. BGUG: Collection of the Botanical Garden University of Ghent.

Actiniopteris australis: Réunion, VIANE 8433.

Adiantum capillus-veneris: Location unknown; BGUG.

Angiopteris hypoleuca: Location unknown; GUBG 1967/0852.

Antrophium giganteum: Réunion; VIANE 8268.

Arachniodes aristata: Location unknown; BGUG.

Asplenium aethiopicum: Uganda: VIANE 11230.

Asplenium affine: Réunion; VIANE 8227.

Asplenium auriculatum: Puerto Rico; VIANE 10602.

Asplenium auritum: Venezuela; VIANE 10256.

Asplenium ceterach: CV405.

Asplenium centrafricanum: Uganda; BELLEFROID 392.

Asplenium compressum: St. Helena; VIANE 8851.

Asplenium cristatum: Puerto Rico; VIANE 10651.

Asplenium currori: Ivory Coast; GUBG 1979/2824.

Asplenium cuspidatum: Venezuela; VIANE 10112.

Asplenium daucifolium: Réunion; VIANE 8445.

Asplenium dregeanum: Uganda; VIANE 11199.

Asplenium exiguum: Mexico; VIANE 11102.

Asplenium friesiorum: Uganda; VIANE 11183.

Asplenium hemionitis: Location unknown; BGUG.

Asplenium juglandifolium: Puerto Rico; VIANE 10667.

Asplenium loxoscaphoides: Uganda; BELLEFROID 340.

Asplenium lunulatum: Rep. South Africa; VIANE 6558.

Asplenium nidus: Myanmar; VIANE 9630.

Asplenium polyodon: Uganda; VIANE 11211.

Asplenium radicans: Puerto Rico; VIANE 10639.

Asplenium ruta-muraria: France; VIANE 10040.

Asplenium rutifolium: Uganda; VIANE 11510.

Asplenium scolopendrium: Belgium; VIANE 10971.

Asplenium serra: Venezuela; VIANE 10222.

Asplenium sertularioides: Uganda; BELLEFROID 405.

Asplenium tenerum: Indonesia; VIANE 9043.

Asplenium tenuicaule: China; VIANE 10888.

Asplenium theciferum: Kenya; BELLEFROID 297.

Asplenium trichomanes: France; VIANE 9566.

Asplenium varians: China; VIANE 10733.

Asplenium vulcanicum: Indonesia; NJO 35.

Athyrium filix-femina: Location unknown; BGUG.

Blechnum brasiliense: Location unknown; BGUG.

Bolbitis heteroclita: Genova; BGUG 1970/0086.

Cheilanthes sp.: Mexico; VIANE 11051.

Cyathea capensis: Rep. South Africa; VIANE 6615.

***Cyclosorus sp.*:** Location unknown; BGUG.

Cystopteris cf. millefolia: Mexico; VIANE 11079.

Danaea elliptica: Puerto Rico; VIANE 10636.

Davallia trichomanoides: Location unknown; BGUG 1970/0085.

Diplazium proliferum: Réunion; VIANE 8281.

Drynaria cf. mollis: China; VIANE 6985.

Dryopteris intermedia: Portugal; VIANE 8940.

Elaphoglossum sp.: Tanzania; VIANE 7718.

Equisetum arvense: Location unknown; BGUG.

Equisetum hyemale: Location unknown; BGUG.

Equisetum ramosissimum: La Palma; VIANE 8118.

Equisetum x littorale: Location unknown; BGUG.

Hymenasplenium excisum: China; VIANE 11263.

Hymenasplenium obscurum: China; VIANE 11286.

Hymenasplenium unilaterale: Réunion; VIANE 8334.

Lepisorus sp.: Uganda; VIANE 11233.

Lonchitis sp. Location unknown; BGUG.

Hupperzia gnidioides: Réunion: VIANE 8481.

Hupperzia squarrosum: Location unknown; BGUG.

Nephrolepis hirsutula: Location unknown; BGUG.

Osmunda vauchelii: China; VIANE 11306.

Pellaea falcata: Location unknown; BGUG 1975/1278.

Platycerium bifurcatum: Location unknown; BGUG.

Polypodium azoricum: Portugal; VIANE 5394.

Polystichum sp.: Venezuela: VIANE 10134.

Psilotum nudum: Location unknown; BGUG.

Pteridium aquilinum: Location unknown; BGUG.

Pteris cretica: Réunion; VIANE 8475.

Pyrrosia sp.: Uganda: VIANE 11223.

Rumohra sp.: Venezuela; VIANE 10104.

Salvinia auriculata: Location unknown; BGUG.

Selaginella grandis: Location unknown; BGUG.

Selaginella kraussiana: Rep. South Africa: VIANE 6566.

Tectaria gemmifera: Kenya: BGUG 75/3306.

Todea barbara: Rep. South Africa; VIANE 11588.

Trichomanes speciosum: Spain; VIANE 5520.

Woodwardia orientalis: Location unknown; BGUG 1900/2729.
